# Severe pulmonary hypertension in aging female apolipoprotein E-deficient mice is rescued by estrogen replacement therapy

**DOI:** 10.1186/s13293-017-0129-7

**Published:** 2017-03-20

**Authors:** Soban Umar, Rod Partow-Navid, Gregoire Ruffenach, Andrea Iorga, Shayan Moazeni, Mansoureh Eghbali

**Affiliations:** Department of Anaesthesiology, Division of Molecular Medicine, David Geffen School of Medicine at UCLA, BH-160 CHS, 650 Charles E Young Dr. South, Los Angeles, CA 90095-7115 USA

**Keywords:** Pulmonary hypertension, Apolipoprotein E, Sex differences, Age, Estrogen

## Abstract

**Background:**

Apolipoprotein E (ApoE) is a multifunctional protein, and its deficiency leads to the development of atherosclerosis in mice. Patients with pulmonary hypertension (PH) have reduced expression of ApoE in lung tissue. ApoE is known to inhibit endothelial and smooth muscle cell proliferation and has anti-inflammatory and anti-platelet aggregation properties. Young ApoE-deficient mice have been shown to develop PH on high fat diet. The combined role of female sex and aging in the development of PH has not been investigated before. Here, we investigated the development of PH in young and middle-aged (MA) female ApoE-deficient mice and explored the role of exogenous estrogen (E2) replacement therapy for the aging females.

**Methods:**

Wild type (WT) and ApoE-deficient female mice (Young and MA) were injected with a single intraperitoneal dose of monocrotaline (MCT, 60 mg/kg). Some ApoE-deficient MA female mice that received MCT were also treated with subcutaneous E2 pellets (0.03 mg/kg/day) from day 21 to 30 after MCT injection. Direct cardiac catheterization was performed terminally to record right ventricular systolic pressure (RVSP). Right ventricular (RV), left ventricular (LV), and interventricular septum (IVS) were dissected and weighed. Lung sections were examined using trichrome and immunofluorescence staining. Western blot analyses of lung and RV lysates were performed.

**Results:**

In WT female mice, the severity of PH was similar between young and MA mice as RVSP was not significantly different (RVSP = 38.2 ± 1.2 in young vs. 40.5 ± 8.3 mmHg in MA, *p* < 0.05). In ApoE-deficient mice, MA females developed significantly severe PH (RVSP = 63 ± 10 mmHg) compared to young females (RVSP; 36 ± 3 mmHg, *p* < 0.05 vs. MA female). ApoE-deficient MA females also developed more severe RV hypertrophy compared to young females (RV hypertrophy index (RV/[LV + IVS]) = 0.53 ± 0.06 vs. 0.33 ± 0.01, *p* < 0.05). ApoE-deficient MA female mice manifested increased peripheral pulmonary artery muscularization and pulmonary fibrosis. E2 treatment of MA female ApoE-deficient mice resulted in a significant decrease in RVSP, reversal of pulmonary vascular remodeling, and RV hypertrophy. In MA female ApoE-deficient mice with PH, only the expression of ERβ in the lungs, but not in RV, was significantly downregulated, and it was restored by E2 treatment. The expression of ERα was not affected in either lungs or RV by PH. GPR30 was only detected in the RV, and it was not affected by PH in MA female ApoE-deficient mice.

**Conclusions:**

Our results suggest that only aging female ApoE-deficient but not WT mice develop severe PH compared to younger females. Exogenous estrogen therapy rescued PH and RV hypertrophy in aging female ApoE-deficient mice possibly through restoration of lung ERβ.

## Background

Pulmonary hypertension (PH) is characterized by arterial obliteration resulting from excessive proliferation of pulmonary vascular smooth muscle and endothelial cells [1]. PH is associated with a progressive elevation in pulmonary arterial pressure leading to right ventricular (RV) hypertrophy and RV failure.

Apolipoprotein E (ApoE) is a multifunctional protein known to reduce circulating oxidized low-density lipoproteins and atherogenesis in the vessel wall [2]. ApoE knockout mice have increased oxidized lipids [3] and develop atherosclerosis on high fat diet [4]. ApoE is also known to inhibit endothelial and smooth muscle cell proliferation [5, 6], key pathologic features of pulmonary vascular disease [7, 8], and has anti-inflammatory and anti-platelet aggregation properties [9]. ApoE deficiency leads to enhanced platelet-derived growth factor signaling, which is important in the pathobiology of PAH [10]. ApoE knockout mice also develop pulmonary hypertension on high fat diet [10]. Interestingly, patients with pulmonary arterial hypertension (PAH) have reduced expression of ApoE in lung tissue [10, 11]. Recently published studies from our group and others have implicated the involvement of oxidized lipids in the development of PH [12–16]. Therefore, ApoE knockout mice represent a model that is susceptible for the development of PH.

Although females are more likely to be diagnosed with some forms of PH [17], they are also protected against PH in different animal models [18–20], a phenomenon known as the “estrogen paradox” of PH [21]. This sex difference in experimental PH has been suggested to be in part due to the protective effects of estrogen, as ovariectomy exacerbates PH and pre-treatment/treatment with estrogen and its metabolites attenuates PH and RV dysfunction [22–27]. Estrous cyclicity peaks at 7–8 months and starts to decline by 9 months of age, with cessation of cyclicity occurring between 13 and 16 months of age in mice [28]. Post-menopausal women have an increased risk for the development of some forms of PH, [29] and hormone replacement therapy prevents the progression of PH in these forms of PH [30]. Other investigators have also suggested that severe PH has now become overwhelmingly a disease of post-menopausal women [20]. In fact, despite higher incidence of the disease in female patients as shown in the REVEAL registry of PH patients, there is a clear shift in the mean age of diagnosis towards older age, particularly in the female patients in the USA [31].

Taken together, the shift of PAH patient population towards post-menopausal women, the decreases of ApoE in human PAH lung tissue, and the susceptibility of ApoE-deficient mice to develop PH, makes ApoE-deficient mice a very interesting model to study the effect of aging on development of PH in females. Here, we compared the effect of aging on severity of PH in ApoE-deficient mice vs. wild type young and MA mice. We also examined the potential for exogenous estrogen replacement therapy for rescuing severe PH in aging female ApoE-deficient mice.

## Methods

### Animals and treatments

Female ApoE-deficient mice (young, 7–8 months old) and middle-aged (MA, 12–13 months old) as well as female C57BL6 wild type (WT) mice (young, 4 months old) and MA (14 months old) were used for the study. We have carefully followed the estrous cycle by checking vaginal smears in middle-aged mice (ApoE-deficient mice and WT mice) for 4–5 consecutive days. All MA mice showed no changes in the estrous cycle, and the majority of the cells were leukocytes and nucleated epithelial cell which is consistent with metestrus and diestrus cytology [32, 33]. So, we confirmed that MA ApoE-deficient or WT female mice that we used for this study were not cycling at around 12–14 months of age. Our finding is in agreement with previous study from Nelson et al. in 1995 showing C57/BL6 mice become acyclic around 12–17 months old [34]. Mice were injected with a single intraperitoneal dose of MCT (60 mg/kg). MCT has been shown to induce PH in mice [35–37]. MCT was dissolved in 1 N HCl, the pH was adjusted to 7.4 and diluted with phosphate buffered saline (PBS) before injection. MCT was injected at day 0 that induced severe PH by ~day 30.

Some MA female ApoE-deficient mice that were injected with MCT were treated with subcutaneous continuous release estrogen (E2) pellets via a subcutaneous 10-day continuous release pellet of 0.03 mg E2/kg/day (Innovative Research of America, E2 group) from day 21 to 30 after MCT. Some MA female ApoE-deficient mice were injected with saline and served as controls (CTRL group). Protocols received institutional review and committee approval.

#### Cardiac and pulmonary hemodynamics

The RVSP was measured directly by inserting a catheter (1.4 F Millar SPR-671, ADInstruments) connected to a pressure transducer (Power Lab, ADInstruments) into the RV just before sacrifice. Briefly, for cardiac catheterization, the mice were anesthetized with a mixture of Ketamine (80 mg/kg) and Xylazine (8 mg/kg) intraperitoneally. The animals were placed on a controlled warming pad to keep the body temperature constant at 37 °C. After a tracheotomy was performed, a cannula was inserted, and the animals were mechanically ventilated. After a midsternal thoracotomy, mice were placed under a stereomicroscope (Zeiss, Hamburg, Germany) and a pressure-conductance catheter (model 1.4 F Millar SPR-671) was introduced via the apex into the RV and positioned towards the pulmonary valve. The catheter was connected to a signal processor (ADInstruments) and RV pressures were recorded digitally. After recording the pressures, heart and lung tissues were removed rapidly under deep anesthesia for preservation of protein integrity.

#### Gross histologic evaluation

The right ventricular (RV) wall, the left ventricular (LV) wall, and the interventricular septum (IVS) were dissected. The ratio of the RV to LV plus septal weight [RV/(LV + IVS)] was calculated as the Fulton index of RV hypertrophy.

#### Western blot analysis

Lungs and RV were homogenized at 4 °C in (mM): 150 NaCl, 50 Tris-HCl, 1 EGTA. 1 EDTA, 1 NaF, 1 PMSF, 1 Na3VO4, 1% NP-40, 0.1% SDS, and 0.5% sodium deoxycholate (pH 7.4) supplemented with protease and phospatase inhibitor cocktails (Roche). The samples were centrifuged at 12,000 g for 10 min, and the supernatants were collected. Protein concentration was measured, and 100 μg of total protein was loaded on a 4–20% gradient Tris-HCl/SDS polyacrylamide gel, electrotransferred to nitrocellulose paper, blocked with 5% non-fat dry milk in 20 mM TBS with 0.1% Tween, and incubated with primary antibodies overnight at 4 °C. Blots were then indirectly labeled using infrared fluorophore conjugated anti-rabbit and anti-mouse secondary antibodies for 1 h and visualized with the Odyssey™ Imaging System (Li-Cor). Equal loading of protein onto each lane in the gel was confirmed by probing for Vinculin. In the immunoblots, all samples were run on the same gel or on two gels at the same time due to the lack of space. The blots were incubated together with the primary and secondary antibodies and were scanned together with the same laser intensity. Two adjacent representative lanes from each group are shown.

#### Immunohistochemistry and imaging

Lungs were fixed in 4% paraformaldehyde (PFA) in 0.1 M Na_2_HPO_4_ and 23 mM NaHPO_4_ (pH 7.4) for 4 h on ice. The tissue was then immersed in ice-cold 20% sucrose in 0.1 M Na_2_HPO_4_ and 23 mM NaHPO_4_ (pH 7.4) overnight to cryoprotect the tissue, mounted using OCT, and transversal 4–6 μm sections were obtained with a cryostat. Tissue sections were stained with immunofluorescence to assess pulmonary vascular remodeling and Masson trichrome stain to assess pulmonary fibrosis. The images were acquired using light microscopes (Axiovert 135, Zeiss, and Nikon Eclipse E 400) or with a laser scanning confocal microscope (Olympus). Pulmonary fibrosis was quantified using a grid that divided the field of view into 100 squares, the number of collagenous tissue (blue stain) in the grid was scored as 1 (present) or 0 (absent). Results are expressed as the percentage occupied by fibrosis to the total area examined.

#### Immunofluorescence staining

Lung sections (4–6 μm) were fixed in acetone for 15 min at –20 °C. The sections were then washed with PBS + 0.1% Triton three times, incubated with 10% normal goat serum in PBS + 0.1% Triton for 30 min to block the background. The sections were then incubated with primary antibodies in PBS + 0.1% Triton + 1% normal goat serum at 4 °C overnight. The sections were then washed with PBS + 0.1% Triton three times, incubated with the appropriate secondary antibodies in PBS + 0.1% Triton + 1% normal goat serum at room temperature for 1 h. After washing the secondary antibodies with PBS + 0.1% Triton three times, the sections were mounted using ProLong Gold (Molecular Probes) for imaging with a laser scanning confocal microscope (Olympus). For assessment of pulmonary arteriolar wall thickness, only distal pulmonary arteries less than 50 μm were quantified (3–4 vessels per mouse). Pulmonary arteriolar wall thickness was calculated by subtracting diameter of the lumen from total diameter of the vessel, divided by total diameter of the vessel. As the diameter of the vessel and lumen are not usually similar in different directions, pulmonary arteriolar wall thickness was measured in two different directions and averaged.

#### Reagents

Primary antibodies used were anti-smooth muscle actin (Thermofisher, 701457, 1:200 dilution), anti ERα (Santa Cruz, Sc-542, 1:1000 dilution), anti ERβ (Thermoscientific, PA1-31013, 1:1000 dilution), GPR30 (Lifespan Bioscience, LS-A4272, 1:1000 dilution), and anti Vinculin (Sigma, V9131, 1:1000 dilution). Secondary antibodies used were anti-rabbit alexa-fluor 594 (Invitrogen, A11012, 1:1000 dilution), goat anti-rabbit-IgG-AlexaFluor680 (Li-Cor, 926-32211, 1:10000 dilution), and goat anti-mouse-IgG-IR Dye800CW (Li-Cor, 926-68070, 1:10000 dilution) for Western immunoblotting.

### Statistical analysis

Student’s *t* test and one-way ANOVA tests were used to compare between groups using SPSS13.0 for Windows. When significant differences were detected, individual mean values were compared by post-hoc tests that allowed for multiple comparisons. *P* < 0.05 was considered statistically significant. Values are expressed as mean ± SEM.

## Results

### In ApoE-deficient mice, young females develop less severe pulmonary hypertension than MA female mice

**Fig. 1:**
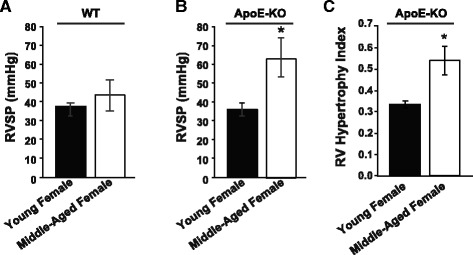
Development of severe PH in middle-aged female ApoE-deficient mice. **a** *Bar graphs* showing right ventricular systolic pressure (RVSP, mmHg) as a marker of severity of PH in in young (*n* = 5) and middle-aged (*n* = 3) WT female mice. **b** *Bar graphs* showing RVSP in young (*n* = 5) and middle-aged (*n* = 4) ApoE-deficient female mice. **c** *Bar graphs* showing right ventricular hypertrophy index (RV/LV + IVS) as a marker of RV hypertrophy in young (*n* = 3) and middle-aged (3) ApoE-deficient female mice. *p < 0.05 vs. young female (*t* test); Values are expressed as mean ± SEM

Since ApoE-deficient mice are more susceptible to development of PH, we compared the severity of PH in WT and ApoE-deficient female mice with aging. In WT female mice, the severity of PH was similar between young and MA as RVSP was not significantly different (RVSP = 38.2 ± 1.2 in young vs. 40.5 ± 8.3 mmHg in MA, *p* < 0.05, Fig. 1a). In ApoE-deficient mice, MA female mice developed significantly worse PH (RVSP = 63 ± 10 mmHg), compared to young females (RVSP; 36 ± 3 mmHg, *p* < 0.05 vs. MA females, Fig. 1b). ApoE-deficient MA females also had more severe RV hypertrophy compared to young females (RV hypertrophy index (RV/[LV + IVS]) = 0.53 ± 0.06 vs. 0.33 ± 0.01, *p* < 0.05, Fig. 1c). These results suggest that MA ApoE-deficient mice develop more severe PH compared to WT mice.

### Increased pulmonary vascular remodeling and pulmonary fibrosis in MA females compared to young female ApoE-deficient mice

**Fig. 2:**
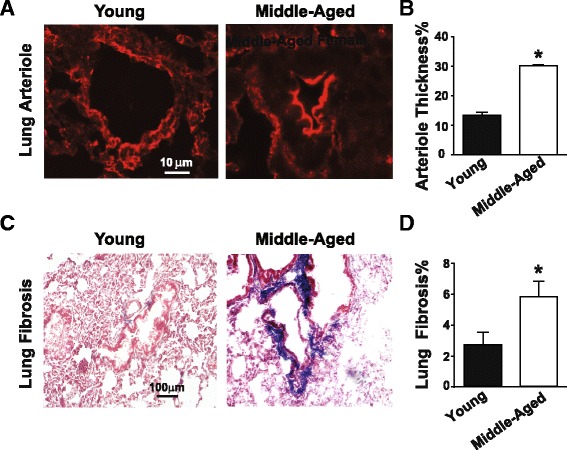
Development of pulmonary vascular remodeling and pulmonary fibrosis in middle-aged female ApoE-deficient mice. **a** Immunofluorescence images showing α-smooth muscle actin stained pulmonary arterioles in young and middle-aged female ApoE-deficient mice. **b** Quantification of arteriolar wall thickness for vessels less than 50 μm in young (*n* = 3) and MA (*n* = 3) ApoE-difiecent mice. *p < 0.05 vs. young female (*t* test). Expressed as mean ± SEM. **c** Masson trichrome stained lung sections showing pulmonary fibrosis (*blue*) in young female and middle-aged female ApoE-deficient mice. **d** Quantification of pulmonary fibrosis in young (*n* = 5) and MA (*n* = 4) ApoE-deficient mice. *p < 0.05 vs. young female (*t* test). Values are expressed as mean ± SEM

ApoE-deficient MA female mice also demonstrated increased pulmonary vascular remodeling compared to young female mice. The pulmonary arteriolar medial hypertrophy in MA female ApoE-deficient mice was significantly higher compared to young female mice (Fig. 2a, b). ApoE-deficient MA female mice also demonstrated increased pulmonary fibrosis compared to young female mice as shown by Masson trichrome staining of lung sections (Fig. 2c, d). These data further support the severity of PH in ApoE-deficient female mice as they age.

### Exogenous estrogen replacement therapy rescues PH in aging female ApoE-deficient mice

**Fig. 3:**
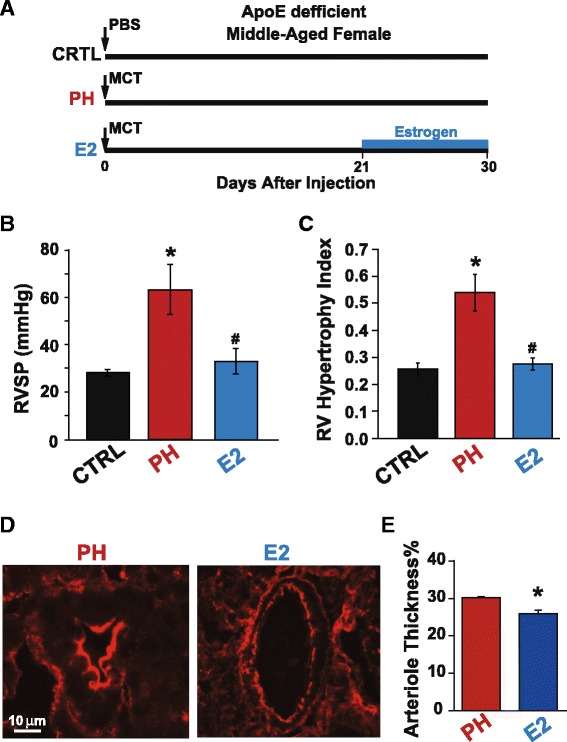
Rescue of PH in middle-aged female ApoE-deficient mice by exogenous estrogen replacement therapy. **a** Experimental protocol showing middle-aged female ApoE-defeicent mice receiving either a single injection of phosphate buffered saline (PBS) for CTRL group at day 0, or single injection of monocrotaline (MCT, 60 mg/kg) for PH group, or single injection of monocrotaline (MCT, 60 mg/kg) at day 0 followed by estrogen (E2) treatment from day 21 to 30 in E2 group. **b**–**c** *Bar graphs* showing RVSP (mmHg), and RV hypertrophy index (Weight ratio of RV/LV + IVS) for ApoE-deficient middle-aged female mice in CTRL (*n* = 4), PH (*n* = 4), and E2 (*n* = 4) treated groups. *p < 0.05 vs. CTRL, #*p* < 0.05 vs. PH. **d** Immunofluorescence images for pulmonary vascular remodeling comparing medial hypertrophy in middle-aged female ApoE-deficient mice of PH group and E2 treated group. **e** Quantification of arteriolar wall thickness for vessels less than 50 μm in MA ApoE-deficient mice in PH (*n* = 3) and in E2 (*n* = 4) groups. *p < 0.05 vs. PH (*t* test). Expressed as mean ± SEM

As ApoE-deficient MA females developed more severe PH compared to young females, we examined whether exogenous estrogen (E2) therapy after establishment of PH could rescue PH in ApoE-deficient mice (Fig. 3a). The RVSP was significantly reduced by exogenous estrogen replacement therapy in MA female ApoE-deficient mice with PH from day 21 to 30 after MCT (63.3 ± 10.5 in PH vs. 33 ± 5.5 mmHg in E2 group, *p* < 0.05, Fig. 3b). In fact, E2 rescued PH in ApoE-deficient MA mice as the RVSP in E2 treated group was not significantly different that MA control group that received PBS (28.3 ± 1.3 mmHg). The RV hypertrophy index was also reversed by E2 therapy (Fig. 3c). E2 treatment also significantly reduced the adverse pulmonary arteriolar hypertrophy (Fig. 3d, e).

### Estrogen treatment restores reduced lung estrogen receptor-beta expression levels in MA females with PH to levels comparable in young females

**Fig. 4:**
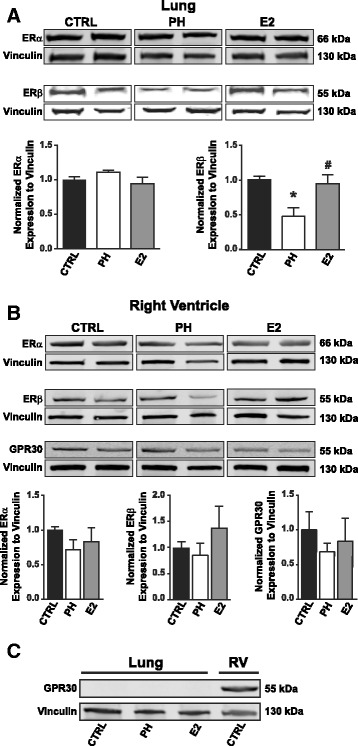
Restoration of estrogen receptor beta expression in the lungs of ApoE-deficient middle-aged female mice with estrogen therapy. **a** Representative Western immunoblot images (*upper*) and *bar graphs* (*lower*) in the lungs from MA ApoE-deficient mice treated with PBS (control, *n* = 4), MCT (PH group, *n* = 3), and MCT with E2 (E2 group, *n* = 5) showing ERα and ERβ protein expression. *p < 0.05 vs. control, #*p* < 0.05 vs. PH (one-way ANOVA). Values are expressed as mean ± SEM. **b** Representative Western immunoblot images (*upper*) and *bar graphs* (*lower*) in RV from MA ApoE-deficient mice treated with PBS (control, *n* = 4), MCT (PH group, *n* = 3), and MCT with E2 (E2 group, *n* = 5) showing ERα, ERβ, and GPR30 expression. Values are expressed as mean ± SEM. **c** Western immunoblot images showing expression of GPR30 only in the RV, but not in the lungs. In all blots Vinculin was used as loading control and expression of each protein was normalized to Vinculin

E2 exerts its biological actions mainly through estrogen receptor alpha (ERα), estrogen receptor beta (ERβ), and GPR30. We examined the expression of estrogen receptors in both lungs and RV in ApoE-deficient MA mice in control, PH, and E2 treated group. In ApoE-deficient MA female mice, the expression of ERα was not altered in the lungs and RV of either CTRL, PH, or E2 treatment groups (Fig. 4a, b). Interestingly, the expression of ERβ in the lungs was significantly reduced in ApoE-deficient MA female mice with PH compared to control (MA CTRL = 1 ± 0.04, MA PH = 0.48 ± 0.11, *p* < 0.05 vs. MA CTRL, Fig. 4a). Estrogen therapy restored the expression of ERβ in the lungs of ApoE-deficient MA females (E2 = 0.95 ± 0.12, *p* < 0.05 vs. MA PH group, Fig. 4a). The expression of ERβ was not affected in the RV of MA ApoE-deficient in PH or by E2 treatment (Fig. 4b). GPR30 was only detected in the RV, and it was not affected by PH or E2 treatment in MA ApoE-deficient mice (Fig. 4b, c).

## Discussion

The shift of PAH patient population towards post-menopausal women, the decreases of ApoE in human PAH lung tissue, and the susceptibility of ApoE-deficient mice to develop PH, makes ApoE-deficient mice a very interesting model to study the effects of aging on development of PH in females. In this study, we first compared the effect of aging on severity of PH in ApoE-deficient mice vs. WT female mice. We discovered differences between young and middle-aged (MA) female mice in the development of severe PH only in ApoE-deficient, but not in WT female mice since ApoE-deficient MA females developed more severe PH compared to young females. Next, we examined whether exogenous estrogen (E2) therapy could rescue established PH in ApoE-deficient MA female mice. We found that exogenous estrogen replacement therapy rescued PH in ApoE-deficient MA female mice after they had cessation of estrous cyclicity. The rescue action of E2 in MA ApoE-deficient mice seems to be linked to lung estrogen receptor beta (ERβ) restoration.

The role of ApoE has been well established in atherosclerosis as ApoE-deficiency results in the development of atherosclerosis in mice [4]. In the context of PH, patients have reduced expression of ApoE in their lungs [11]. ApoE has also been shown to act in an axis along with two regulators of smooth muscle cell proliferation and migration, the bone morphogenetic protein 2 (BMP-2) and peroxisome proliferator-activated receptor γ (PPARγ) [38–40]. In fact, loss-of-function-mutations in the BMP receptor II (BMPRII) are linked to the development of PH [41].

ApoE-deficient mice represent a vulnerable mouse strain for the development of pulmonary vascular disease and may serve as a good model for studying the influences of age on PH in females. Young ApoE-deficient mice have been shown to develop PH on high fat diet in a sex specific manner [10]. These sex differences were attributed to insulin resistance and testosterone’s ability to inhibit the secretion of adiponectin (a vasoprotective adipocytokine) in the adipocytes of male mice. Thus, elevated levels of adiponectin in female ApoE-deficient mice accounted for the less severe vascular phenotype. Treatment of male ApoE-deficient mice with rosiglitazone (a PPARγ agonist) resulted in higher plasma adiponectin levels and a complete regression of PH [10]. Recent studies from our lab and others have implicated the involvement of oxidized lipids in the development and progression of PH [12–14, 42–44]. Hemnes et al. recently provided evidence for RV lipotoxicity in heritable PAH [45]. Abnormalities in fatty acid metabolism can be detected in the blood and myocardium in human PAH and are associated with in vivo cardiac steatosis and lipotoxicity. Murine data suggested that lipotoxicity may arise from reduction in fatty acid oxidation [46]. Impaired fatty acid oxidation and increased expression of the lipid transporter CD36 are the key mechanisms underlying lipid deposition in the BMPR2 mutant RV, which were exacerbated in the presence of dietary lipids [47].

We used the already vulnerable ApoE-deficient mice as a model to study the influence of increasing age on the development of PH in females. Although females are more likely to be diagnosed with some forms of PH [17], they are also protected against PH in different animal models [18–20], a phenomenon known as the “estrogen paradox” of PH [21]. This sex difference in experimental PH has been suggested to be in part due to the protective effects of estrogen, as ovariectomy exacerbates PH and pre-treatment/treatment with estrogen and its metabolites attenuates PH and RV dysfunction [22–27]. The cardioprotective effects of estrogen in experimental models of PH are well documented [48–50]. Previously, we have shown that estrogen rescues pre-existing severe PH in rats by restoring lung and RV structure and function that is maintained even after cessation of estrogen therapy [24]. Estrogen also protects RV function in the SuHx model of PAH in mice directly by stimulating RV contractility and indirectly by protecting against pulmonary vascular remodeling, underscoring the therapeutic potential of estrogen in PAH [51]. Estradiol improves RV function in rats with severe angioproliferative PH, suggesting significant RV-protective estrogen receptor-mediated effects of estrogen [27]. Female SuHx rats exhibited superior cardiac index than SuHx males. Ovariectomy worsened SuHx-induced decreases in cardiac index and SuHx-induced increases in RV hypertrophy and inflammation. E2 repletion in ovariectomized rats attenuated SuHx-induced increases in RVSP, RV hypertrophy, and pulmonary artery remodeling and improved cardiac index and exercise capacity. Furthermore, E2 repletion ameliorated SuHx-induced alterations in RV glutathione activation, proapoptotic signaling, cytoplasmic glycolysis, and proinflammatory cytokine expression [27]. On the other hand, estrogen metabolite 16α-hydroxyestrone has been shown to exacerbate Bone Morphogenetic Protein Receptor Type II (BMPR II) associated PAH [52]. In addition, PAH has been associated with dysregulated estrogen and serotonin signaling. Overexpression of the serotonin transporter in mice results in an estrogen-dependent development of PAH [53]. The estrogen paradox in pulmonary hypertension still exists and more research is needed to explain the molecular basis of this paradox [21].

The effect of menopause on the disease severity and progression in PH has not been extensively investigated. Post-menopausal women with increasing age have been shown to be at increased risk for the development of certain types of PH [29]. The progression of PH in these patients was prevented by hormone replacement therapy [30] suggesting possible endogenous estrogen depletion and a potential role of exogenous estrogen replacement therapy. In our study, we also observed worsening of PH with increasing age only in ApoE-deficient, but not in WT female mice. The worsening of PH in only ApoE-deficient MA female mice points towards the possible interplay between advancing age, cessation of estrous cyclicity, and increasing oxidized lipids in these mice. This is not surprising as ApoE is known to inhibit endothelial and smooth muscle cell proliferation [5, 6] and has anti-inflammatory and anti-platelet aggregation properties [9]. ApoE-deficiency leads to enhanced platelet-derived growth factor signaling, which is important in the pathobiology of PAH [10]. Exogenous estrogen replacement therapy resulted in complete rescue of cardiopulmonary hemodynamics and pulmonary vascular remodeling associated with PH in the ApoE-deficient MA female mice. As mentioned earlier, others have attributed sex differences in the development of PH in female ApoE-deficient mice to elevated levels of adiponectin as female ApoE-deficient mice exhibited less severe vascular phenotype [10]. On the other hand, in aging female ApoE-deficient mice, the successful rescue of PH by exogenous estrogen replacement suggests endogenous estrogen depletion as a main factor, although a potential contributory role for adiponectin cannot be ruled out.

Estrogen exerts most of its biological effects via its receptors, ERα, ERβ, and GPR30. ERβ has been implicated in the protective effects of estrogen against experimental PH in rats [24]. ERα has pro-proliferative properties in certain types of cancers whereas ERβ exerts anti-proliferative effects [54]. The anti-hypertrophic properties of E2 in the heart are mediated mainly via ERβ [24, 55]. We observed a downregulation of ERβ in the lungs of aging female ApoE-deficient mice that was restored by exogenous estrogen replacement therapy, coinciding with lowering of RVSP and decrease in pulmonary arteriolar medial thickness. The expression of ERα was not affected both in lungs and RV in PH. GPR30 was only detected in the RV, and it was not affected by PH or by E2 treatment in ApoE-deficient MA female mice.

## Conclusions

In conclusion, middle-aged ApoE-deficient female mice develop more severe PH compared to younger female ApoE-deficient mice possibly due to the interplay between oxidized lipids, and cessation of estrous cyclicity, as a result of menopause. Exogenous estrogen therapy rescues PH in aging female ApoE-deficient mice likely through lung estrogen receptor beta repletion.

## Abbreviations

ApoE: Apolipoprotein E
BMP-2: Bone morphogenetic protein 2
CTRL: Control
E2: Estrogen
ERα: Estrogen receptor alpha
ERβ: Estrogen receptor beta
GPR30: G protein coupled receptor 30
IVS: Interventricular septum
LV: Left ventricle
MA: Middle-aged
MCT: Monocrotaline
PAH: Pulmonary arterial hypertension
PH: Pulmonary hypertension
PPARγ: Peroxisome proliferator-activated receptor γ
RV: Right ventricle
RVSP: Right ventricular systolic pressure
SuHx: Sugen hypoxia
